# RNABP COGEST: a resource for investigating functional RNAs

**DOI:** 10.1093/database/bav011

**Published:** 2015-03-16

**Authors:** Sohini Bhattacharya, Shriyaa Mittal, Swati Panigrahi, Purshotam Sharma, Preethi S. P., Rahul Paul, Sukanya Halder, Antarip Halder, Dhananjay Bhattacharyya, Abhijit Mitra

**Affiliations:** ^1^Center for Computational Natural Sciences and Bioinformatics (CCNSB), International Institute of Information Technology (IIIT-H), Gachibowli, Hyderabad 500032, and ^2^Computational Science Division, Saha Institute of Nuclear Physics (SINP), 1/AF Bidhannagar, Kolkata 700064, India; Present address: Purshotam Sharma, Department of Chemistry and Biochemistry, University of Lethbridge, Lethbridge, Alberta, T1K4B8, Canada.; Present address: Rahul Paul, University of Texas Medical Branch, 301 University Blvd., Research Building 17, Routing no: 1156, Galveston, TX 77555, USA.

## Abstract

Structural bioinformatics of RNA has evolved mainly in response to the rapidly accumulating evidence that non-(protein)-coding RNAs (ncRNAs) play critical roles in gene regulation and development. The structures and functions of most ncRNAs are however still unknown. Most of the available RNA structural databases rely heavily on known 3D structures, and contextually correlate base pairing geometry with actual 3D RNA structures. None of the databases provide any direct information about stabilization energies. However, the intrinsic interaction energies of constituent base pairs can provide significant insights into their roles in the overall dynamics of RNA motifs and structures. Quantum mechanical (QM) computations provide the only approach toward their accurate quantification and characterization. ‘RNA Base Pair Count, Geometry and Stability’ (http://bioinf.iiit.ac.in/RNABPCOGEST) brings together information, extracted from literature data, regarding occurrence frequency, experimental and quantum chemically optimized geometries, and computed interaction energies, for non-canonical base pairs observed in a non-redundant dataset of functional RNA structures. The database is designed to enable the QM community, on the one hand, to identify appropriate biologically relevant model systems and also enable the biology community to easily sift through diverse computational results to gain theoretical insights which could promote hypothesis driven biological research.

**Database URL:** http://bioinf.iiit.ac.in/RNABPCOGEST

## Introduction

There is a remarkable growth in the ranks of newly discovered non-(protein)-coding RNAs (ncRNAs) (1–7) that regulate diverse cellular processes. This has been accompanied by a rapid increase in efforts toward characterizing and understanding their complex structures and functionalities, at the molecular level. This has also given rise to the question of how RNA molecules, with only four different nucleotides, are capable of exhibiting such great structural complexity and functional diversity. RNA 3D structures are known to be stabilized by a large number of non-canonical (nc) base pairs (8–10) of which at least 200 variants have been either identified or postulated. The variation in the physicochemical properties of these base pairs may hold clues to the observed structural and functional diversity. Hence, we observe a lot of activity in two distinct research fields: quantum mechanical (QM) computations of interaction energies in nucleic acids and RNA structural bioinformatics (11).

The intrinsic interaction energies and stabilities of constituent base pairs of large RNA 3D motifs have been attractive targets for QM calculations to understand diverse functional dynamics of the motifs. Although the intrinsic interaction energies constitute only one of several complex physicochemical factors that govern the variation in shapes and functional roles of different base pairs, the importance of these interaction energies is underlined by the fact that QM computations provide the only approach toward their accurate quantification and characterization (11). Such QM studies have already revealed many important features of base pairing, such as pyramidalization of exocyclic amino groups (12), possibilities of geometrical substates of base pairs involving amino acceptor interactions (13–15), base pairing involving ribose O2’ as hydrogen bond donor and/or acceptor (13–17), importance of base phosphate interactions (18), base pairing involving bifurcated and water-mediated hydrogen bonds, role of protonation in base pairing (19, 20), characterization of C-H···O/N hydrogen bonds in base pairing, etc.

Several of these base-pairing features have highlighted the importance of QM calculations for the proper interpretation of experimental data and in refining X-ray crystallographic data.

At the same time, structural bioinformatics of RNA has evolved in response to the rapidly accumulating evidence that ncRNAs, which constitute a major proportion of the transcriptome, play critical roles in gene regulation and expression. Important developments in this area include the identification and characterization of the nc base pairs, higher order structures and recurrent 3D motifs involving them. Not only has this given rise to a widely accepted framework for the classification of base pairs in terms of their geometries (21), the concomitant evolution of the concept of isostericity (22) has established the importance of base pair shapes (and not simply their identities) as a major determinant in molecular recognition processes involving RNA. The concept of isostericity, in turn, has also introduced an added dimension aiding the process of RNA sequence alignment.

As has been pointed out in recent literature (11), the two research fields, quantum mechanics and bioinformatics, have a great potential to complement each other to provide insights into the physicochemical basis of RNA structure, dynamics, function and evolution. However, they appear to be largely separated in the current literature. We feel that one of the main reasons for this is the apparent paucity of communication between the corresponding scientific communities.

Structural bioinformatics relies heavily on known 3D structures and structural motifs. Thus, most of the currently available RNA structure databases like RNA STRAND (23), FRABASE (24), FR3D RNA motif Atlas (25), SCOR (26), RNA Junction database (27), RNA CoSSMos (28) and most recently the RNA Bricks (29) database, provide information about either secondary structures or motifs. Few databases which provide exclusive information about nc base pairs are NCIR (30), BPS (31), FRABASE (24) and FR3D RNA base pair catalogue available through webFR3D (32, 33). In order to contextually correlate base pairing geometries with 3D RNA structures, these databases mainly focus on crystal occurrences of different base pair types, their structural annotation and geometric descriptions. However, they do not provide any direct information about energies. On the other hand, there is an exponential growth in the volume of QM analysis data on base pair stabilities, involving advanced post-Hartree-Fock QM theories (34, 35) or DFT-based theories (36–39). It is possibly due to the absence of ready availability of comprehensive resources, that their use in the analysis of RNA functional mechanisms appears to be very limited. Our perception regarding the utility of a ready reference for geometries, energies and stabilities of nc RNA base pairs is also vindicated by the favorable response to ‘Non-canonical RNA Base Pair Database’ which was made available earlier to the scientific community, by some of the contributing authors of this article. The ‘Benchmark Energy and Geometry Database’ (40) which, to our knowledge, is the only other database that provides interaction energy related information, covers only some selected base pairs and stacks. RNA Base Pair Count, Geometry and Stability (RNABP COGEST) addresses this issue of effective communication across domains, by annotating and systematically organizing validated QM analysis data, in a manner relevant for understanding functionalities of ncRNA at the molecular level.

## Materials and methods

### Base pair annotation

Each nucleotide base has three distinct interacting edges—Watson Crick (W) edge, Hoogsteen (H) (C-H edge in pyrimidine) edge and Sugar (S) edge. Each of the edges of any particular base consists of distinct set of hydrogen bond donors and acceptors. Unlike double helical DNA which is characterized by canonical A-T/U and G-C base pairs, a variety of nc base pairs are observed in RNA, as single-stranded RNA molecule folds on itself and open up possibilities for the constituent nucleotide bases to interact with others through any of their three distinct edges in either *cis* or *trans* glycosidic bond orientation. Leontis and Westhof (21) have classified all base pairing geometries into 12 distinct geometric families and given a comprehensive nomenclature scheme which is followed here to annotate base pair entries in this database.

### QM calculation data

Most of the QM data stored here pertains to isolated base pairs, which are modeled by complete or partial removal of the backbone atoms. In some cases ribose C1’ atom is replaced by a methyl group or a hydrogen atom. In some other cases the ribose sugar is fully retained, while the phosphate group is replaced by a hydroxyl group. It may be noted that the intrinsic interaction energies, corresponding to these models, need to be used carefully while explaining the role of different base pairs in actual biological context. Further, while most data refer to interaction energies calculated in vacuum, solvent phase calculation data are also reported wherever available. All entries are accompanied by clear documentation of the model details and the level of QM theory used for the computations.

### Data resources for optimized base pair geometries

Information related to ground state optimized geometry of all types of canonical and nc base pairs, viz., base pair parameters, detailed hydrogen bonding descriptions and interaction energy along with its components as calculated using different QM methods, are mostly collected from the published literature (13–17, 19, 41–45). Unpublished data computed by the contributing authors have been used to obtain some additional information in some cases. The images of optimized base pairs are generated using Pymol from the available coordinates of the optimized geometries.

### Data resources for occurrence frequencies

To calculate occurrence frequencies of each base pair type, a non-redundant dataset of 167 pdb files is considered to avoid statistical bias. These 167 pdb files are obtained from non-redundant dataset available in HD-RNAS database (46). HD-RNAS classifies all the RNA structures available in PDB according to their functional classes, e.g. transfer RNA (tRNA), messenger RNA (mRNA), ribosomal RNA (rRNA), ribozymes, riboswitches, ribonucleases, etc. (http://www.saha.ac.in/biop/www/HD-RNAS.html). Obviously, the tRNA and ribosomal RNA are further classified according to the amino acid it may carry and sedimentation coefficient, respectively. In order to exclude small synthetic RNA constructs, we have used a resolution cut-off of 3.5 Angstrom or better and chain length cut-off of 30 nucleotides or larger for the classification procedure. The structures are then further classified according to the source organism from which the RNA molecules were isolated and crystallized. The non-redundant dataset consists of the best representative RNA structures from each of these sub-classes, determined by the best resolution and *R*-factor (free *R*-value). Base pairing interactions present in the above-mentioned dataset are extracted by using BPFIND software (47). The dataset used here is expected to provide meaningful glimpses of base pair occurrences and their structural contexts in functional RNA molecules. For each instance of occurrence of a query base pair, the E_value (47) and the six base pair parameters (buckle, propeller, open, stagger, shear and stretch) (48) are reported as calculated by BPFIND (47) and NUPARM (49) softwares, respectively.

### Database design

RNABP COGEST is a relational database developed in MYSQL (version 5.5.35). A website using the PHP programming language (version 5.3.10), HTML5, CSS3, JavaScript and JQuery constitutes the front end. The user interface provides forms, with appropriate fields for building complex queries, which are executed using PHP scripts running on the server-end of the MySQL platform in an Apache2.0 system. The results are viewable as HTML tables in a dynamically generated page, displayed in the user's browser.

## Result and discussion

### Scope of the database

RNABP COGEST contains information about nearly 200 different nc base pairs including protonated base pairs, base pairs with amino acceptor interactions, water-mediated base pairs and also modeled or predicted base-pairing geometries. Through a user friendly web interface, available at http://bioinf.iiit.ac.in/RNABPCOGEST, users can navigate to retrieve the required information. Here we have extensively linked the information available in the initial database ‘Non-canonical RNA Base-pair database’ available at http://www.saha.ac.in/biop/www/db/local/BP/rnabasepair.html as a companion database. The data available in the present database are listed below.

#### Basic information on optimized base pairs

This section includes proper annotation of base pair family according to the Leontis–Westhof (LW) scheme (21), the isostericity subclass (22) and the source pdb from which the particular base pair is taken for optimization. Multimodality is observed in hydrogen bonding pattern for certain base pairs within some of their geometries. This gives rise to optimized base pairs, which have same LW names, but different interaction energies. The different hydrogen bonding patterns are indicated using roman numeric within parenthesis, immediately following the corresponding base pair name. For example in W:W Trans family U:U(I) and U:U(II) show different hydrogen bonding patterns and therefore have different stability.

#### Geometry

A quantitative description of base pair arrangements in 3D space is provided for each base pair type, in terms of six intra base pair parameters (buckle, propeller, open, stagger, shear and stretch) (48), which define mutual orientation of two interacting bases, and E_value (47), which defines the goodness of a base pair in terms of average values of all hydrogen bonding distances and angles. The parameters mentioned are available both for ground state optimized geometries and for all instances of crystal structure occurrences. Depending on the availability of raw data, we provide coordinates and 2D images of all the optimized geometries with an option for download. For some base pairs, the pre-optimized geometries are also provided.

#### Root-mean-square deviation (RMSD)

Generally, due to the physicochemical effects of neighboring interactions, the crystal geometries can be different from their local energy minima. RMSD between the experimental and optimized structures and also between optimized structures using different QM theories are included, where available, along with their superposed structures to estimate the structural change upon optimization. This information sometimes provides meaningful insights into the contextual effect on a particular base pair.

#### Interaction energy

The information about intrinsic interaction energy and its components such as electrical, exchange repulsion, charge transfer, etc., obtained for all optimized geometries using energy decomposition analysis (50, 51) are collated from the existing literature in a systematic manner. For some base pair geometries ‘prior to optimization’ interaction energy data are also available.

#### Hydrogen bonding

Description of specific hydrogen bonding network for all optimized geometries, in terms of hydrogen bond donors and acceptors, their mutual distances and donor–hydrogen–acceptor angles, is made available. Hydrogen bonding patterns, observed in their corresponding pre-optimized geometries, wherever available, are also reported.

### Database features

#### Guided browsing

**Figure 1.**
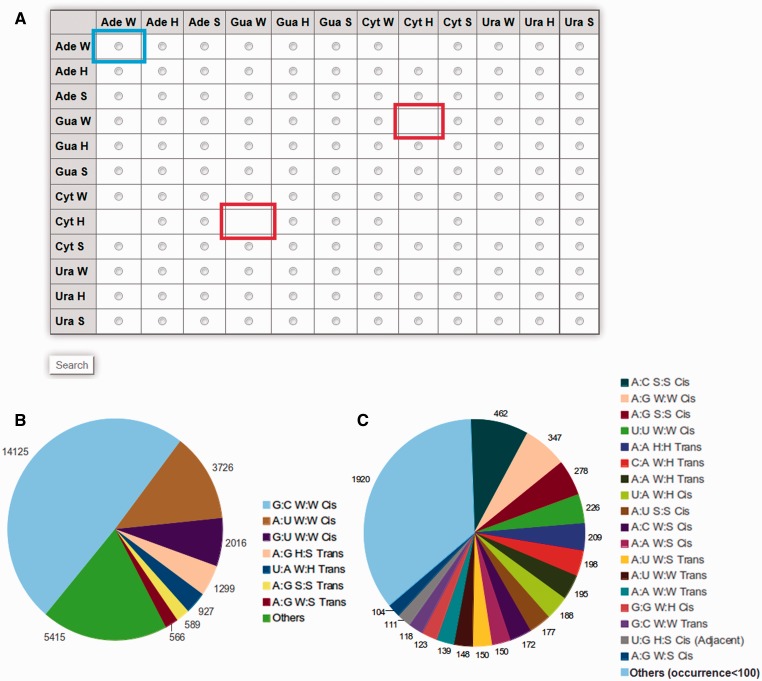
(**A**) Matrix table for efficient browsing of base pair information available in RNABP COGEST. Each cell of the matrix indicates a specific base–base interaction in a particular geometry, e.g. the cell highlighted by the blue box indicates W:W type interaction between two Adenine bases. Selecting the radio button of a cell, followed by clicking on the search button at the end of the table, opens up a list of all possible base pairs between the given bases in their selected geometries. Cells without radio buttons (example cells marked in red) correspond to base pairs and geometries which cannot be stabilized through hydrogen bonds. (**B**) Pie chart showing distribution of occurrence frequencies of seven ‘most frequent’ base pairs in the non-redundant dataset. (**C**) Distribution of occurrence frequencies of base pairs, referred to as ‘others’ in (B), and, which occur more than 100 times in the non-redundant dataset.

Detailed information about specific query base pairs can be obtained by selecting from the matrix table displayed on the browse page available at http://www.bioinf.iiit.ac.in/RNABPCOGEST/home_browse.html. The matrix table displays individual radio buttons for all possible edge-to-edge base pairing combinations. Boxes, without radio buttons, imply unavailability of those particular base pairs in the database. A pie chart, depicting relative occurrences of different base pairs, is also provided beside the matrix table (Figure 1), to guide users in identifying frequently occurring base pairs while browsing.

#### Advanced search options

Multiple search options are provided on the search page: http://wwwbioinf.iiit.ac.in/RNABPCOGEST/home_search.html. The quick search option allows the user to uniquely specify a particular base pair, e.g. a particular base pair A:C W:W *Cis* with protonated (A) can be specified from the options given in drop down lists to directly access all information related to its count, geometry and stability. Alternatively, data regarding sets of base pairs, sorted on the basis of occurrence frequencies, energy parameters, structural parameters, hydrogen bond types and various other characteristics such as protonation site, water mediation, involvement of amino acceptor interactions, etc., can be accessed easily using the user friendly dropdown menus and search boxes to create complex queries in real time. In all of these search options, users can specify one or more parameters with a desired value or a range of value. The searching procedures and the search results are explained elaborately the help page available at http://bioinf.iiit.ac.in/RNABPCOGEST/help.html.

#### User guidance

Keeping in mind the interdisciplinary nature of the potential user group, an elaborate ‘Help’ page and a ‘Terms and Definitions’ page have been incorporated. They have been designed to facilitate researchers from the domain of biology to not only conveniently navigate through the data, but also to provide relevant web links to enable non-QM specialists to evaluate the relevance of the QM data in a domain context.

### Application of the database

RNABP COGEST is designed to build an effective communication across domains by putting together a comprehensive annotated resource which could enable the QM community to identify appropriate model systems which are of interest to biologists. It would also enable the biology community to easily sift through a vast array of computational results to identify theoretical insights which could promote hypothesis-driven biological research. In the previous section, it has been mentioned that different structural features of RNA molecules can be explained by QM computational studies. Some of the yet unanswered questions, which can be addressed by computational studies, include the occurrence of highly distorted or ‘far away from local minima’ geometries in experimental RNA structures, the role of modified base pairs compared with their respective unmodified regular canonical and nc base pairs, the involvement of nc base pairs in stacking, etc. This database is designed to facilitate this process.

**Figure 2.**
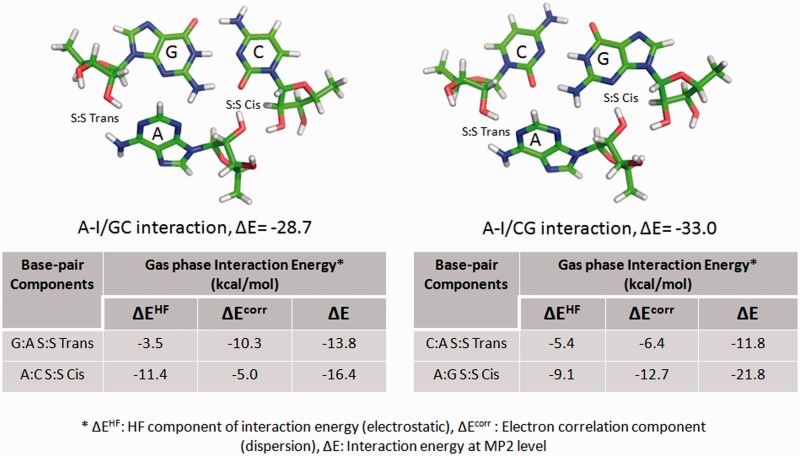
Intrinsic interaction energy of A-I/CG triad (top right) is higher than that of A-I/GC triad (top left), though the latter shows greater stability during simulation. This may be explained (cf. text) in terms of the variation in contribution of the correlation term (dispersion) for G:A S:S Trans base pair in the A-I/GC triad, and that for the C:A S:S Trans base pair in the A-I/CG triad. Images and table are adapted from Réblová et al. (52).

In 2011, Réblová et al. (52) explained the apparently contradictory observations related to MD simulation and QM stabilities, respectively, in different A-minor interactions present in kink-turn motifs. As explained in Figure 2, it was observed that a motif which was intrinsically most stable as estimated by gas phase QM calculations either changes its interaction geometry or breaks down in the course of simulations. Authors rationalize this apparent anomaly by invoking the balance between electrostatic (HF term) and dispersive (electron correlation term) components of interaction energy. RNABP COGEST provides a fast and comprehensive access to annotated energetics data which could facilitate our understanding of the behavior of this and other similar cases. For example, a recent study on Sarcin-Ricin internal loop motif (SR-motif) reports two structurally observed variants of the reference native SR-motif (variant 0), characterized by isosteric substitutions of two different A:G H:S Trans base pairs, respectively. Variant 1 involves substitution of the A18:G4 H:S Trans base pair by C18:C4 H:S Trans, and variant 6 involves substitution of the A8:G15 H:S Trans base pair by A8:A15 H:S Trans (53) (see Supporting information S1 for details). In course of MD simulation, the C18:C4 base pair, in the flexible region of variant 1, showed far greater geometric fluctuations compared with the corresponding A18:G4 base pair in the variant 0. This extra bit of noticeable fluctuation was characterized by switching between H:S Trans, water-mediated W:W *Cis* and intermediate water-mediated ‘near H:S Trans’ geometries of the C18:C4 base pair. In contrast, the A8:A15 H:S Trans base pair in the G bulge region of the variant 6 displayed very little fluctuation similar to that of the corresponding A8:G15 base pair in variant 0. Interestingly, intrinsic interaction energies, calculated at the B3LYP/6-31G(d,p)//RI-MP2/aug-cc-pVD level, show that both the mutant base pairs in the two variants, respectively, have significantly lower interaction energies (−11.6 kcal/mol for C:C H:S Trans and −10.2 kcal/mol for A:A H:S Trans) compared with that of the corresponding A:G H:S Trans base pairs (−15.1 kcal/mol) present in the variant 0. Whereas, this conveniently explains why the C:C H:S Trans shows higher fluctuations in MD simulation, it is indeed counterintuitive that the A:A H:S Trans pair with even lower interaction energy does not show any enhancement in fluctuation compared with the reference native pair. From RNABP COGEST we can observe that the intrinsic stability of C:C H:S trans base pair is dominated by the electrostatic component (HF term, 73.3% of total interaction energy). In contrast, the dispersion component (electron correlation, 54.9% of total interaction energy) dominates in case of the A:A H:S Trans base pair. The higher proportion of the electrostatic component may be responsible for the enhanced fluctuation observed in the C:C H:S trans base pair in variant 1. It is pertinent to mention here that replacement of A:G with C:C involves a substitution of a purine–purine base pair by a pyrimidine–pyrimidine base pair in variant 1. It is possible that such a substitution may adversely affect the stacking energies, and thus, also contribute toward the increased flexibility. An accurate estimation of these effects is however beyond the scope of this article.

The potential applications of such a database is also exemplified in some of our recent work investigating the structural variation and stability of double helical RNA fragments containing multiple nc base pairs (54, 55) and in our attempts to correlate sequence in terms of di-nucleotide steps and stacking overlaps (56, 57).

Geometry and stability-related information of different base pairs should also be useful in the emerging area of RNA tectonics for designing new RNA constructs (58) and to develop nucleic acid based nano-devices (59). The advanced search options also open up possibilities for the identification of unusual base pairs which need further investigations.

Another niche area which this database could cater to is related to MD simulations of large biologically relevant systems, not only in terms of extracting MM force field parameters, but also in terms of facilitating QM-MM-based simulations.

## Future work

The current release (version 1.0, release date: 23 October 2014) includes data associated with all observed and predicted base pairing geometries for which data are available in literature. Since we have extracted information from different resources, complete information for all the base pairs is not available. To collate the maximum amount of available information for all base pair types and also in the interests of uniformity of data, a number of fields are kept empty. One of our future targets is to fill these gaps. Another important target is to incorporate the information about the structural context of occurrences of each base pair types, which may provide insights about their functional roles. Along with the regular update of the database with newly published relevant data, we plan to implement more complex search options involving Boolean queries.

## Supplementary Data

Supplementary data are available at *Database* Online.

Supplementary Data
