# Supplementary material for: RNABP COGEST: a resource for investigating functional RNAs
Source: Database (Oxford). 2015 Mar 16;2015:bav011. doi: 10.1093/database/bav011 (PMC4360618; doi:10.1093/database/bav011)
Supplement: Supplementary Data [file supp_2015_bav011_index.html]

RNABP COGEST: a resource for investigating functional RNAs — Supplementary Data 

# RNABP COGEST: a resource for investigating functional RNAs

## Supplementary Data

files

**Files in this Data Supplement:**

- Supplementary Data - doc file
