# Supplementary material for: RNABP COGEST: a resource for investigating functional RNAs
Source: Database (Oxford). 2015 Mar 16;2015:bav011. doi: 10.1093/database/bav011 (PMC4360618; doi:10.1093/database/bav011)
Supplement: Supplementary Data [file supp_bav011_S1.doc]

**Supplementary information for**

**RNABP COGEST: A Resource for Investigating Functional RNAs**

Sohini Bhattacharya^1^, Shriyaa Mittal^1^, Swati Panigrahi^2^, Purshotam Sharma^1,3^, Preethi S.P^1^, Rahul Paul^2,4^, Sukanya Halder^2^, Dhananjay Bhattacharyya^2^,* and Abhijit Mitra^1^,*

^1^Center for Computational Natural Sciences and Bioinformatics (CCNSB), International Institute of Information Technology (IIIT-H), Gachibowli, Hyderabad 500032, India

^2^Computational Science Division, Saha Institute of Nuclear Physics (SINP), 1/AF, Bidhannagar, Kolkata 700064, India

^3^*Present address:* Department of Chemistry and Biochemistry, University of Lethbridge, Lethbridge, Alberta, T1K4B8, Canada,

^4^*Present address:* 301 University Blvd., Research Building 17, Routing no: 1156, University of Texas Medical Branch, Galveston, Tx: 77555

**Supplementary figure S1.**


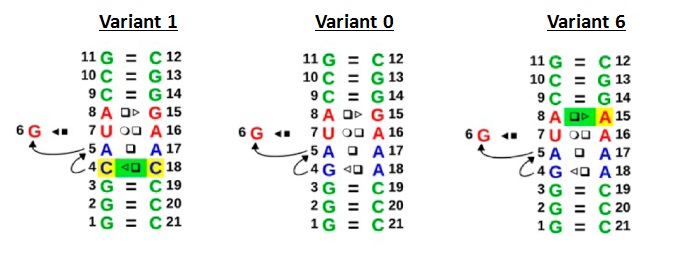


Figure S1: Three structurally observed variants of Sarcin-Ricin internal loop motif. Canonical stems, G bulge region and flexible regions are shown in green, red and blue respectively. Mutated bases in different variants are highlighted in yellow boxes and the corresponding base pairing geometries are highlighted in green boxes. In variant 1, 4/18 A:G H:S Trans base pair in flexible region is mutated to isosteric C:C H:STrans base pair and in variant 6, 8/15 A:G H:S Trans base pair in G-bulge region is mutated to another isosteric A:A H:S trans base pair. Here we are considering this example because in both the cases one A:G H:S Trans base pair is changing to an isosteric base pair, but showing different fluctuation in MD simulation run. The figure is taken from reference (53) mentioned in the original paper.
